# Tuberculosis and pharmacological interactions: A narrative review

**DOI:** 10.1016/j.crphar.2020.100007

**Published:** 2020-12-15

**Authors:** Niccolò Riccardi, Diana Canetti, Paola Rodari, Giorgio Besozzi, Laura Saderi, Marco Dettori, Luigi R. Codecasa, Giovanni Sotgiu

**Affiliations:** a StopTB Italia Onlus, Milan, Italy; b Department of Infectious - Tropical Diseases and Microbiology, IRCCS Sacro Cuore Don Calabria Hospital, Negrar di Valpolicella, Verona, Italy; c Clinic of Infectious Diseases, IRCCS San Raffaele Scientific Institute, Milan, Italy; d Clinical Epidemiology and Medical Statistics Unit, Department of Medical, Surgical and Experimental Sciences, University of Sassari, Sassari, Italy; e Regional TB Reference Centre, Villa Marelli Inst., Niguarda Hospital, Milan, Italy

**Keywords:** Tuberculosis, Drug-drug interactions, Clinical use, TB treatment

## Abstract

Even if major improvements in therapeutic regimens and treatment outcomes have been progressively achieved, tuberculosis (TB) remains the leading cause of death from a single infectious microorganism. To improve TB treatment success as well as patients' quality of life, drug-drug-interactions (DDIs) need to be wisely managed. Comprehensive knowledge of anti-TB drugs, pharmacokinetics and pharmacodynamic (PK/PD) parameters, potential patients’ changes in absorption and distribution, possible side effects and interactions, is mandatory to built effective anti-TB regimens. Optimization of treatments and adherence to international guidelines can help bend the curve of TB-related mortality and, ultimately, decrease the likelihood of treatment failure and drop-out during anti-TB treatment. Aim of this paper is to describe the most relevant DDIs between anti-TB and other drugs used in daily clinical practice, providing an updated and “easy-to-use” guide to minimize adverse effects, drop-outs and, in the long run, increase treatment success.

## Highlights

• Tuberculosis (TB) remains the leading cause of death from a single infectious microorganism.
• Comprehensive knowledge of anti-TB drugs and PK/PD parameters is mandatory to built effective anti-TB regimens.
• Drug-drug-interactions (DDIs) need to be avoided and/or wisely managed to ensure treatment success.
• Optimization of anti-TB treatment to avoid DDIs can help to bend the curve of TB related mortality.

## 1 Introduction

Even if major improvements in therapeutic regimens and treatment outcomes have been progressively achieved, tuberculosis (TB) remains the leading cause of death from a single infectious microorganism (World Health Organization, 2014, 2019).

Since the introduction of rifampicin in the 70s’, an effective short-course regimen for drug-susceptible (DS) forms of TB has been saving millions of lives worldwide. A standardized regimen characterized by a 2-month intensive (bactericidal) phase with four drugs [isoniazid (H), rifampicin (R), ethambutol (E), and pyrazinamide (Z); 2HRZE] and a 4-month continuation (sterilizing) phase with HR is key to achieve microbiological and clinical cure, if taken correctly (World Health Organization, 2014, 2019).

However, 10 million individuals are estimated to develop TB annually worldwide and treatment success is hampered by drug-resistant strains of *Mycobacterium tuberculosis* (Mtb), comorbidities, scarce adherence to treatment, adverse events of anti-TB drugs, and drug-drug interactions (DDIs) (Villa et al., 2020; Riccardi et al., 2019; Bisson et al., 2020; Soanker et al., 2020). Optimization of treatments and adherence to international guidelines can help bend the curve of TB-related mortality, as well as decrease the likelihood of treatment failure and drop-out during anti-TB treatment (Nahid et al., 2019). New drugs have been added to the anti-TB armamentarium and new DDIs can occur (Berger et al., 2020; Dheda et al., 2019; Riccardi et al., 2018; Bigelow et al., 2020).

Aim of this paper is to describe the most relevant DDIs between anti-TB and other drugs used in daily clinical practice, providing an updated and “easy-to-use” guide to minimize adverse effects, drop-outs and, ultimately, increase treatment success.

## 2 Methods

For the first-line drugs HRZE and for every TB drugs enlisted in the World Health Organization guidelines for the management of DR-TB (WHO consolidated guidelines, 2019), the following information are described: i) pharmacokinetics and pharmacodynamic (PK/PD) parameters; ii) potential changes in absorption and distribution; ii) most prevalent DDIs; iv) therapeutic drug monitoring (TDM) when available.

### 2.1 Anti-TB drugs

#### 2.1.1 Rifampicin

Rifampicin, which belongs to the ansamycins and was discovered in 1965 from the soil bacteria *Amycolatopsis rifamycinica* (Sensi, 1983), displays antibacterial activity against Gram-positive cocci and Gram-negative bacteria, as well as against mycobacteria (Rothstein, 2016). It can selectively inhibit the DNA-dependent RNA polymerase both by sterically blocking the path of the elongating RNA at the 5′ end and by decreasing the affinity of the RNA polymerase for short RNA transcripts (Campbell et al., 2001; Schulz and Zillig, 1981).

Rifampicin is an highly lipid-soluble drug with a PK profile that shares equal distribution in plasma and tissues, with a plasmatic half-life ranging between 2 and 5 ​h it strongly induces human P450 cytochrome oxidases, notably CYP3A4, CYP2A, CYP2B, CYP2C, and CYP3A, as well as the human P glycoprotein ABC transporter, thus leading to a number of remarkable DDIs (Riccardi et al., 2020a) (Table 1).

**Table 1:** Most common rifampicin DDIs.

Class of drug	Drug	Mediated protein or mechanism	Rifampicin PL	Drug PL	Other effects	References
Analgesics	Methadone	CYP3A4; CYP2B6; CYP2C19; CYP2C9		↓	overdose if inducer discontinuation	Kreek et al. (1976)
	Morphine	hepatic metabolism induction				Fromm et al. (1997)
	Oxycodone, fentanyl, codeine	CYP3A4			overdose if inducer discontinuation	Product Information. OxyContin (oxycodone)
Anesthetics	Alfentanil	CYP3A4		↓		–
	Halothane				hepatotoxicity, hepatic encephalopathy	Most and Markle (1974)
Antacids	Aluminum hydroxide/magnesium hydroxide	increased gastric pH; chelation	↓			Product Information. Rifadin (rifampin)
Anti-arrhythmics	Amiodarone	CYP3A4		↓		Zarembski et al. (1999)
	Disopyramide	–				Aitio et al. (1981)
	Propafenone	CYP3A4				Dilger et al., 2000, Castel et al., 1990
	Digitoxin	CYP2C19; CYP3A4; P glycoprotein				–
	Digoxin	P glycoprotein; renal tubular secretion				Greiner et al., 1999, Novi et al., 1980
	Quinidine	CYP3A4				Bussey et al. (1984)
Antibiotics	Clarithromycin	CYP3A4	↑	↓		Wallace et al. (1995)
	Clindamycin	CYP3A4		↓		Bernard et al. (2015)
	Cefazolin, other cephalosporins	vitamine K-dependent coagulopathy			severe coagulation disorders	Lerner and Lubin (1974)
	Chloramphenicol	hepatic metabolism induction		↓		Prober (1985)
	Dapsone	CYP3A34; CYP2C9; CYP2E1				Horowitz et al., 1992, Peters et al., 1977
	Doxicicline	CYP3A4				Colmenero et al. (1994)
	Linezolid	P glycoprotein				Egle et al., 2005, Gebhart et al., 2007, Gandelman et al., 2011
	Moxifloxacin	glucuronidation; sulphation; P glycoprotein				Nijland et al. (2007)
Anticoagulants	Warfarin	CYP3A4		↓		Casner (1996)
	Dabigatran	P glycoprotein				Product Information. Pradaxa (dabigatran).
	Apixaban	CYP3A4; P glycoprotein				Product Information. Eliquis (apixaban)
	Rivaroxaban	CYP3A4				Product Information. Xarelto (rivaroxaban)
	Edoxaban	P glycoprotein				Product Information. Savaysa (edoxaban)
Anticonvulsants	Phenytoin	CYP2C9; CYP2C19		↓		Kay et al. (1985)
	Lamotrigine	glucuronidation				Wimpelmann et al., 2019, Ebert et al., 2000
Antidepressants	Nortriptyline, amytriptiline	CYP450		↓		Bebchuk and Stewart (1991)
Anti-diabetics	Chlorpropamide	CYP450		↓		–
	Rosiglitazone	CYP2C8				Niemi et al. (2004)
Antiemetics	Ondansetron	CYP3A4; CYP1A2		↓		Villikka et al. (1999)
Antifungals	Caspofungin	–		↓		Product Information. Cancidas (caspofungin)
	Fluconazole	CYP450				Tett et al., 1992, Rajasingham et al., 2018
	Itraconazole, ketoconazole	CYP3A4	↑			Tucker et al. (1992)
	Posaconazole	CYP3A4; P glycoprotein; UGT1A1	↑			Product Information. Noxafil (posaconazole)
Anthelmintics	Praziquantel	CYP450		↓		Ridtitid et al. (2002)
Antimalarials	Atovaquone	rifampicin enzyme-induction	↑	↓		Product Information. Mepron (atovaquone)
	Quinine	CYP3A4		↓		Pukrittayakamee et al. (2003)
Antipsychotics	Haloperidol	CYP3A4		↓		–
Antituberculars	Isoniazid	additive hepatotoxic effect			hepatotoxicity	Acocella et al. (1972)
	Pyrazinamide					McNeill et al. (2003); CDC (2001)
	Delamanid	CYP3A4		↓		EMA, 2013
Antivirals anti-HCV	Daclatasvir, simeprevir, ​sofosbuvir, ledipasvir	CYP3A4; P glycoprotein		↓		Talavera Pons et al. (2017)
Anxiolytics/hypnotics	Zolpidem	CYP3A4; CYP1A2		↓		Villikka et al. (1997)
	Diazepam, triazolam	hepatic metabolism induction				
Bronchodilators	Theophylline	CYP3A4; CYP1A2		↓		Boyce EG, 1985; Powell-Jackson et al. (1985)
Cancer therapies	Cyclophosphamide	CYP3A4; CYP2B6; CYP2C9		↕		Rose BD, 2005
	Irinotecan	CYP3A4		↓		Product Information. Camptosar (irinotecan)
	Lorlatinib				hepatotoxicity	Product Information. Lorbrena (lorlatinib)
	Dasatinib, zanubrutinib					Mu S., 2020
	Tamoxifen, toremifene					–
Contraceptives	Ethinyl estradiol	CYP3A4		↓		Skolnick et al. (1976)
Corticosteroids	Prednisolone, prednisone	–	↓	↓		Lee KH, 1993
	Dexamethasone	–				–
Hypertensives	Losartan	CYP3A4; CYP2C9		↓		Williamson KM, 1998
	Metoprolol, propranolol, bisoprolol	CYP450				Kirch et al. (1986)
	Diltiazem	CYP3A4				–
	Verapamil					–
	Nifedipine					Tada et al. (1992); Holtbecker N, 1996
Immunosuppressants (SOT)	Tacrolimus	CYP3A4; P glycoprotein		↓		Bhaloo and Prasad (2003)
	Cyclosporine		↑			Van Buren et al. (1984)
Lipid lowering drugs	Simvastatin, fluvastatin	CYP2C9		↓		Scripture and Pieper (2001)
	Pravastatin	cMOAT; MRP2				Kyrklund et al. (2004)
Protonic pump inhibitors	Omeprazole, esomeprazole	CYP3A4; CYP2C19		↓		Product Information. Prilosec (omeprazole)
Retrovirals	Zidovudine	glucuronidation		↓		Gallicano et al. (1999)
	Tenofovir alafenamide	P glycoprotein				Custodio JM, 2017; Cerrone M, 2018
	NNRTI	CYP3A4				–
	Efavirenz				hepatotoxicity	Brennan-Benson P, 2005
	PI					MMWR, 2000; CDC, 1996
	Atazanavir		↕			–
	Darunavir/ritonavir					–
	Saquinavir/ritonavir	CYP3A4; P glycoprotein			hepatotoxicity	–
	Raltegravir	UGT1A1				–
	Bictegravir	–				Custodio JM, 2018
	Dolutegravir	UGT1A1; CYP3A4				Dooley K, 2018
Thyroid hormones	Levothyroxine, liothyronin	CYP450		↓		Nolan SR, 1999

Abbreviations: DDI, drug-drug interactions; PL, plasmatic level; SOT, solid organ transplantation; NNRTI, Non-nucleoside reverse transcriptase inhibitors; PI, Protease inhibitors.

It is available both in oral (best adsorbed during fasting, avoiding food interactions) or intravenous formulations (Rothstein, 2016; Gengenbacher and Kaufmann, 2012).

TDM of rifampicin should be ideally assessed 2 ​h post dose on empty stomach, with a desirable range of 8–15 ​mg/L, in order to ensure efficacy and avoid toxicity (Fernandes et al., 2017).

#### 2.1.2 Isoniazid

Isoniazid, firstly discovered in 1920 and used as antimycobacterial compound in the 1950s, displays mycobactericidal action against replicating mycobacteria, whereas it is bacteriostatic against mycobacteria in the latent form (Harvey et al., 2006; Timmins and Deretic, 2006). Its efficacy relies on the destruction of the mycobacterial cell wall, acting as a prodrug activated by the mycobacterial enzyme KatG and generating reactive oxygen species (ROS) that interfere with the mycobacterial cell wall (Timmins and Deretic, 2006; Stehr et al., 2015).

Isoniazid can be administered orally, intramuscularly, or intravenously; it is promptly absorbed after oral administration and reaches the peak of serum concentrations after 0.5–2 ​h (Timmins and Deretic, 2006).

Isoniazid inhibits the cytochrome P450 system and i acts as a mild monoamine oxidase inhibitor (MAO-I) (Stehr et al., 2015) (Table 2).

**Table 2:** Most common isoniazid DDIs.

Class of drug	Drug	Mediated protein or mechanism	Isoniazid PL	Drug PL	Other effects	References
Alcohol antagonists	Disulfiram	dopamine metabolism inhibition			neuropsychiatric disorders	Whittington HG, 1969
Analgesics	Acetaminophen	CYP2E1		↑	hepatotoxicity	Nolan CM, 1994; Crippin JS, 1993
Antacids	Aluminum hydroxide	↓ isoniazid absorption	↓			Hurwitz A, 1974
Anticonvulsants	Carbamazepine	CYP3A4		↑		Wright JM, 1982
	Valproate	hepatic metabolism inhibition		↑	↑ isoniazid toxicity	Dockweiler U, 1987
	Phenitoin	CYP450		↑		Witmer DR, 1984
Anti-diabetics	Oral hypoglycemics and insulin	interference with glucose metabolism			↕ glycemia at drug suspension	Ganguly RJ, 2018
Antifungals	Ketoconazole, itraconazole	CYP3A4		↓		Product Information. Nizoral (ketoconazole)
Antituberculars	Rifampicin	CYP450		↑	hepatotoxicity; haematological abnormalities	Askgaard DS, 1995; Nagayama N, 2004
	Cycloserine	–			dizziness	Mattila MJ, 1969
Bronchodilators	Theophylline	CYP3A4; CYP1A2		↑		Hoglund P, 1987
Recreational drugs	Alcohol	–			hepatotoxicity	Product Information. INH (isoniazid)

Abbreviations: DDI, drug-drug interactions; PL, plasmatic level; NNRTI, Non-nucleoside reverse transcriptase inhibitors; PI, Protease inhibitors.

TDM should be assessed 2 ​h post dose on empty stomach, with a desirable range of 3–6 ​mg/L (Fernandes et al., 2017).

#### 2.1.3 Pyrazinamide

Pyrazinamide is a nicotinamide analog included in the anti-TB regimen in 1970 and characterized by sterilizing activity and efficacy against semi-dormant MTB strains. Moreover, it displays synergistic activity when added to rifampicin-containing regimens (Gopal et al., 2019; Jiménez del Cerro and Rivera Hernández, 1992). In fact, it penetrates necrotic caseous tissue, where it is converted to the active form pyrazinoic acid, killing non-growing, drug-tolerant tubercle bacilli through the inhibition of the coenzyme A biosynthesis (Jiménez del Cerro and Rivera Hernández, 1992).

Pyrazinamide displays excellent oral absorption, without any food interferences (Jiménez del Cerro and Rivera Hernández, 1992) It can increase blood ureic acid level, leading to acute gout. Liver is probably involved in its katabolism.

Concomitant administration of pyrazinamide with isoniazid and/or rifampin is associated with an increased risk of hepatotoxicity due to an additive effect. Careful assessment is needed when it is prescribed with potentially hepatotoxic agents (e.g., pexidartinib, pretomanid, mipomersen).

A unique case of cyclosporine plasmatic level reduction after pyrazinamide association was reported (Peets et al., 1965).

TDM should be ideally assessed 2 ​h post dose on empty stomach, with a desirable range of 20–60 ​mg/L (Fernandes et al., 2017).

#### 2.1.4 Ethambutol

Ethambutol, which was discovered in 1961, is a bacteriostatic against actively growing mycobacterial bacilli: it inhibits the enzymes arabinosyltransferases involved in the synthesis of mycobacterial cell wall (Lee and Nguyen, 2020).

Ethambutol can be administered orally or intravenously and is promptly absorbed after oral administration, reaching the peak of serum concentrations after 2 ​h (Lee and Nguyen, 2020); it undergoes partial hepatic metabolism.

Coadministration with aluminium salts should be avoided because it delays and reduces the absorption of ethambutol (Riccardi et al., 2020b). Other drugs potentially causing optic neuritis should be avoided (Riccardi et al., 2020b).

TDM should be ideally assessed between 2 and 6 ​h post dose on full or empty stomach, with a desirable range of 20–60 ​mg/L (Fernandes et al., 2017).

#### 2.1.5 Linezolid

Linezolid is a synthetic oxazolidinone with bacteriostatic activity against mycobacteria and Gram-positive bacteria (Dryden, 2011). It binds to the 50S subunit of the prokaryotic ribosome, preventing formation of the initiation complex and inhibiting protein synthesis (Quinn and Stern, 2009). Linezolid, which is available both intravenously and orally with excellent bioavailability (fatty food can decrease its absorption) (Dryden, 2011), is metabolized to two inactive metabolites, an aminoethoxyacetic acid (metabolite A) and a hydroxyethyl glycine (metabolite B), with a plasmatic elimination half-life of 3.4–7.4 ​h (Quinn and Stern, 2009). It is a reversible inhibitor of monoamine oxidases A and B and serotonin agonists should be carefully prescribed to avoid the occurrence of the serotonin syndrome (Ziganshina et al., 2013) (Table 3).

**Table 3:** Most common linezolid DDIs.

Class of drug	Drug	Mediated protein or mechanism	Linezolid PL	Drug PL	Other effects	References
Adrenergic agents	Pseudoephedrine	↑ sympathomimetic effect			systolic hypertension	Hendershot PE, 2001
	Phenylpropanolamine					
Antibiotics/antituberculars	Rifampin	P glycoprotein	↓			Trittler R, 2005; Egle H, 2005; Gebhart BC, 2007; Gandelman K, 2011
Anticoagulants	Warfarin	–			low grade ↓ INR	Sakai Y, 2015
Antidepressants		MAOi inhibition			serotonin syndrome	Boyer EW, 2005
SSRI	Paroxetine					
SSRI	Fluvoxamine					
SSRI	Fluoxetine					
SSRI	Citalopram					
SSRI	Fluvoxamine					
SSRI	Sertraline					
SSRI	Escitalopram					
SSRI	Vilazodone					
SNRI	Venlafaxine, desvelafaxina					
SNRI	Duloxetine					
Anti-Parkinson	Rasagiline	MAOi inhibition			serotonin syndrome	Boyer EW, 2005; FDA Drug Safety Communication, 2011
Morphine derivatives	Dextromethorphan	↑ sympathomimetic effect			serotonin syndrome	Hendershot PE, 2001
Opioid analgesics	Meperidine	brainstem 5-HT1A and 2A receptors hyperstimulation			serotonin syndrome	Product Information. Meperidine Hydrochloride Injection, USP (meperidine)
	Fentanyl					Corsini Campioli C, 2020

Abbreviations: DDI, drug-drug interactions; PL, plasmatic level; SSRI, Selective Serotonine Reuptake Inhibitor; SNRI, Serotonine and Norepinephrine Reuptake Inhibitor; MAOi, monoamine oxidase inhibitor.

TDM of linezolid should be ideally assessed immediately before the administration, with a desirable range of 2–7 ​mg/L (Fernandes et al., 2017).

#### 2.1.6 Fluoroquinolones (Levofloxacin and Moxifloxacin)

Fluoroquinolones, which can inhibit the DNA gyrase responsible for supercoiling of nucleic acid, show a broad-spectrum antimicrobial activity (Reynolds et al., 1996). Levofloxacin and moxifloxacin are recommended by the WHO for the treatment of multi-drug resistant (MDR)-TB (WHO consolidated guidelines, 2019). Levofloxacin is available both orally and intravenously, whereas moxifloxacin can be administered only orally. Their prescription with or without other drugs was associated with the risk of cardiac arrhythmias, fungal or bacterial infections, psychosis, and convulsions (Gler et al., 2012) (Table 4).

**Table 4:** Most common antitubercular fluoroquinolones DDIs.

Class of drug	Drug	Mediated protein or mechanism	Levo/moxi PL	Drug PL	Other effects	References
Antacids	Aluminum/magnesium/calcium salts	chelation	↓			(Nix et al., 1990)
	Sucralfate					(Lehto et al., 1994)
Anti-arrhythmics	Quinidine, procainamide (IA class)	↑ QT interval			rare: arrhythmias, torsade de pointes	(Owens, 2001)
	Amiodarone, sotalole (III class)				rare: arrhythmias, torsade de pointes	(Owens, 2001)
Antibiotics/antituberculars	Rifampin	glucuronidation; sulphation	↓ moxifloxacine			(Nijland et al., 2007)
Anticoagulants	Warfarin	metabolism inhibition; vitamin K-producing intestinal bacteria inhibition			↑ INR	Jones CB, 2002
Anti-diabetics	Insulin	ATP-sensitive K channels			↕ glycemia	Gajjar DA, 2000
	Oral hypoglycemics				↕ glycemia	Gajjar DA, 2000
Bronchodilators	Theophylline	GABA?			SNC toxicity	Segev S, 1988
Calcium channel blockers	Bepridil	↑ QT interval			rare: arrhythmias, torsade de pointes	
Cancer therapies	Osimertinib	↑ QT interval			rare: arrhythmias, torsade de pointes	Bian S, 2020
Immunosuppressants (SOT)	Cyclosporine	low grade ↓ hepatic metabolism		↑		Federico S, 2006
	Tacrolimus					Federico S, 2006
NSAIDs	Diclofenac, others	GABA?			CNS toxicity	Segev S, 1988
	Fenbufen		↑		CNS toxicity	Segev S, 1988
Retrovirals	Didanosine	chelation (Al or Mg-containing formulations)	↓			Sahai J, 1993
Supplements	Calcium supplements	chelation	↓			Frost RW, 1992
	Iron supplements					Polk RE, 1989
	Zinc supplements					Polk RE, 1989
Others	Cimetidine	low grade ↓ tubular secretion	↑			Stass H, 2005
	Probenecid					
	Vegetable charcoal	oral moxifloxacin ↓ absorption	↓ moxifloxacine			

Abbreviations: DDI, drug-drug interactions; PL, plasmatic level; Levo, levofloxacin; Moxi, moxifloxacin; NSAIDs, Nonsteroidal anti-inflammatory drugs; SOT, solid organ transplantation; CNS, central nervous system; GABA, gamma-aminobutyric acid receptors.

TDM should be ideally assessed 2 ​h after their administration on full or empty stomach, with a desirable range of 8–12 ​mg/L and 3–5 ​mg/L for levofloxacin and moxifloxacin, respectively (Fernandes et al., 2017).

#### 2.1.7 Delamanid

Delamanid is a dihydro-nitroimidazooxazole with early bactericidal activity for patients aged ≥3 years (WHO consolidated guidelines, 2019; Gupta et al., 2016; Gupta et al., 2015; Matsumoto et al., 2006). It was recently approved for the treatment of MDR-TB.

The pro-drug is activated by a deazaflavin dependent nitro-reductase into a metabolite which blocks the cell wall synthesis of methoxymycolic and ketomycolic acids, two components of mycobacterial (European Medicines Agency, 2014).

Delamanid administered orally has a three-fold increased bioavailability intaking a high-fat meal (Diacon et al., 2011). Its maximum plasmatic concentration is reached 4–5 ​h after oral administration, its half-life lasts 38 ​h, with a steady-state achieved after 10–14 days (Paccaly et al., 2012).

Being a CYP3A4 substrate, its level is strongly reduced in case of co-administration with strong CYP3A4 enzyme inducers (e.g., rifampicin). Lopinavir/ritonavir increase plasmatic levels of delamanid and, then, the risk of toxicity (Riccardi et al., 2020c). It was recommended caution when prescribed with clofazimine (Yadav et al., 2016).

The risk of QTc prolongation is increased when administered with fluoroquinolones and in hypoalbuminemic patients. Currently, no standardized TDM range has been proposed.

#### 2.1.8 Clofazimine

Clofazimine is a hydrophobic riminophenazine which presumably interferes with the mycobacterial respiratory chain and ion transporters (Riccardi et al., 2020c).

It is prescribed for non-tuberculous mycobacteria-related diseases, TB, and leprosy; its oral bioavailability is ∼70%, improved by food for an increased absorption (Riccardi et al., 2020c).

Clofazimine can inhibit CYP3A4 in vitro, but also can weakly induce CYP3A4 (Riccardi et al., 2020c). It can prolong QTc interval and impair the liver function (Riccardi et al., 2020c).

TDM should be assessed 2 ​h after its administration on full or empty stomach, with a desirable range of 0.5–4 ​mg/L (Fernandes et al., 2017).

#### 2.1.9 Bedaquiline

Bedaquiline is an oral diarylquinoline, approved for pulmonary MDR-TB in adults (Yadav et al., 2016; Andries et al., 2014). It blocks the proton pump (specifically, subunit c) for mycobacterial ATP synthase, critically reducing ATP level and, then, causing cell death (Hartkoorn et al., 2014). Synthesis of subunit c is encoded by the atpE gene and its mutation in *Mycobacterium tuberculosis* is directly associated to poor drug susceptibility (Centers for Disease Contr). Furthermore, mutations of the drug efflux pump are linked to bedaquiline resistance, similarly to its upregulation which favours cross-resistance between bedaquiline and clofazimine (Nimmo et al., 2020). Drug susceptibility test (DST) and whole genome sequencing (WGS) for bedaquiline should be performed to assess the susceptibility of the collected strains (Alffenaar et al., 2015). DDIs have been observed between bedaquiline and CYP3A4 inducers and inhibitors (Table 5).

**Table 5:** Most common bedaquiline DDIs.

Class of drug	Drug	Mediated protein or mechanism	Bedaquiline PL	Other drug PL	Other effects	References
Antidepressant	citalopram				QTc prolongation	Drug package leaflet
	escitalopram					
Antibiotic	clarithromycin				QTc prolongation	
	azithromycin					
	levofloxacin					
	moxifloxacin					
	lefamulin					
Antiparasitic	fexinidazole				QTc prolongation	
	piperaquine					
	chloroquine					
	halofantrine					
	pentamidine					
Antifungal	posaconazole	QTc prolongation, inhibition of CYP3A4	↑			
	voriconazole	QTc prolongation, inhibition of CYP3A4	↑			
	fluconazole	QTc prolongation, inhibition of CYP3A4	↑			
	itraconazole	inhibition of CYP3A4	↑			
Antipsychotic	thioridazine				QTc prolongation	
	flupentixol					
	pimozide					
	quetipine					
	amisulpride					
	clozapine					
	aloperidol					
	droperidol					
	olanzapine					
	risperidone					
Cancer drugs	nilotinib				QTc prolongation	
	entrectinib					
	ribociclib					
	vemurafenib					
	dasatinib					
	encorafenib					
	enzalutamide					
	gilteritinib					
	inotuzumab					
	midostaurin					
	mitotane					
	osimertinib					
	toremifene					
	dabrafenib	QTc prolungation, induction of CYP3A4	↓			
	lorlatinib	induction of CYP3A4	↓			
	pexidartinib	induction of CYP3A4	↓			
Procynetic/antiemetic	domperidon				QTc prolongation	
	ondansetron					
Anti-hyperlipidemic	probucol				QTc prolongation	
Anti-arrhythmics	amiodarone				QTc prolongation	
	dronedarone					
	flecainide					
	pilsicainide					
	propafenone					
	dofetilide					
	sotalol					
Antihistamine	astemizole				QTc prolongation	
Antituberculars	clofazimine				QTc prolongation	
	demalanid					
	rifampicin	induction of CYP3A4	↓			Svensson 2015
	rifabutine	induction of CYP3A4	↓			
	rifapentine	induction of CYP3A4	↓			
Antihypertensive	lofexidine	QTc prolongation				
Antiretroviral	lopinavir	inhibition of CYP3A4	↑			(Pandie et al., 2016)
	atazanavir	inhibition of CYP3A4	↑			
	darunavir	inhibition of CYP3A4	↑			
	cobicistat	inhibition of CYP3A4	↑			
	ritonavir	inhibition of CYP3A4	↑			
	efavirenz	induction of CYP3A4	↓			
	etravirine	induction of CYP3A4				
Analgesic	methadone	QTc prolongation				
Pulmonary hypertension drug	bosentan	induction of CYP3A4	↓			
Antiepileptic	cenobamate	induction of CYP3A4	↓			
	carbamazepine	induction of CYP3A4	↓			
	phenobarbital	induction of CYP3A4	↓			
	phenytoin	induction of CYP3A4	↓			
Antivirals anti-HCV	ombitasvir	inhibition of CYP3A4	↑			
	paritaprevir	inhibition of CYP3A4	↑			
	dasabuvir	inhibition of CYP3A4	↑			

Abbreviations: DDI, drug-drug interactions; PL, plasmatic level.

Bedaquiline can cause QT prolongation, leading to cardiac arrhythmia and/or death. Hence, patients should be monitored for symptoms of cardiac toxicity and by electrocardiogram during the follow-up. It should be interrupted in case of clinically significant ventricular arrhythmia or QTc >500 ​ms (Nguyen et al., 2018). Ongoing studies are evaluating the most accurate TDM values (Nguyen et al., 2018; Bolhuis et al., 2016).

#### 2.1.10 Cycloserine/Terizidone

Cycloserine, which is similar to the amino acid D-alanine, can interrupt the inclusion of the D-alanine into the peptidoglycan of the cell wall (Cycloserine, 2008; http://www.tbdrugmonograpa). It has an oral bioavailability when not administered with a high fat meal (http://www.tbdrugmonograpa). It can show central nervous system (CNS) toxicity (careful administration in case of alcohol exposure, history of seizure, depression, suicidal behaviours, and mood instability) and can interfere with the absorption of isoniazid (http://www.tbdrugmonograpa). Cycloserine should be used with extreme caution in patients with renal impairment and avoided when creatinine clearance of <50 ​ml/min (http://www.tbdrugmonograpb). Terizidone, which is composed by two molecules of cycloserine, shares the same pharmacological features of cycloserine, but it can be administered in patients with a creatinine clearance <50 ​ml/min and in patients in dialysis with an appropriate dose adjustment (http://www.tbdrugmonograpa). Terizidone also penetrates less in CNS being therefore more tolerable.

#### 2.1.11 Meropenem/imipenem-cilastatin

Meropenem and imipenem/cilastatin are broad-spectrum carbapenems used with clavulanic acid in the treatment of MDR-TB. Meropenem with clavulanic acid has bactericidal activity and can sterilise cultures within 2 weeks inhibiting the BlaC beta-lactamase (De Lorenzo et al., 2013). They are administered intravenously and may have neuro-toxicity (caution in patients with TB meningitis and when coadministered with ganciclovir and valproic acid). Renal function should be periodically checked due to their renal excretion (De Lorenzo et al., 2013).

TDM of meropenem has a desirable range of 8–12 ​mg/L (Fernandes et al., 2017).

#### 2.1.12 Amikacin

The aminoglycoside amikacin can inhibit mycobacteria, Gram-negative bacteria, *Nocardia* spp., and *Staphylococcus aureus*, by blocking the 30S ribosomal subunit with the modification of the conformation of the A site, reducing proofreading capabilities of the ribosome and thus increasing mistranslation (Ramirez and Tolmasky, 2017).

It is mainly administered intravenously, intramuscularly, through nebulization, but it can be administered intrathecally or intraventricularly (Ramirez and Tolmasky, 2017). It shows a renal excretion, and can increase the risk of ototoxicity and nephrotoxicity, especially in case of long-term exposure. TDM can reduce the risk of adverse events (Ramirez and Tolmasky, 2017). DDIs can be relevant with other drugs associated with oto- and nephron-toxicity (e.g., diuretics, cephalosporins, ciclosporin, colistimethate sodium, and tacrolimus). There is increased risk of hypocalcaemia when prescribed with bisphosphonates.

TDM should be ​< ​5 ​mg/L (trough, immediately before infusion) and 25–35 ​mg/L (peak, 1 ​h after intravenous administration) (http://www.tbdrugmonograpb).

#### 2.1.13 Ethionamide/Prothionamide

Ethionamide, which was discovered in 1959, is a prodrug undergoing hepatic metabolism. Its efficacy was proved for *M. tuberculosis*, *M. bovis, M. Laepre, M. Avium*, *and M. smegmatis* (Ethionamide, 2008; http://www.tbdrugmonograpc). It disrupts the mycobacterial cell wall through the inhibition of the inhA gene product enoyl-ACP reductase. Ethionamide is available orally and can be administered both with and without food (Ethionamide, 2008). Neurotoxicity is linked to increased blood level of ethionamide. Alcohol exposure can favour psychotic reactions. Prothionamide is a thioamide interchangeable with ethionamide (http://www.tbdrugmonograpc).

It should be administered with caution in patients with liver failure. Moreover, it is structurally similar to methimazole and, then, thyroid function should be routinely checked to avoid the occurrence of hypothyroidism (http://www.tbdrugmonograpc). In diabetic patients glucose blood level should be monitored for the risk of hypoglycaemia (http://www.tbdrugmonograpc).

#### 2.1.14 P-aminosalicylic acid (PAS)

PAS, discovered in 1944, is available in an oral formulation; it should be administered with acid food to increase its absorption (Abulfathi et al., 2020).

DDIs with dichlorphenamide may lead to increased levels of PAS by unknown mechanism. PAS may decrease blood level of rifampicin. Moreover, PAS decreases effects of benazepril by pharmacodynamic antagonism and increases adverse events of dapsone.

## 3 Discussion

The ambitious WHO goal of TB elimination can be achieved if a comprehensive strategy is implemented. The WHO TB Strategy, approved by the World Health Assembly in 2014, is built on three pillars (World Health Organization, 2014).

One of them, which can be found in the previous WHO strategy, is based on the improvement of the clinical management of individuals infected by *Mycobacterium tuberculosis* strains (World Health Organization, 2014).

The mismanagement of patients with TB disease can be associated to a poor prognosis, increased risk of transmission of Mtb to susceptible individuals, and emergence (and spread) of drug-resistant strains (Bisson et al., 2020; Nahid et al., 2019; Dheda et al., 2019).

The successful outcome of the TB patients depends on patient- and healthcare-related factors. The appropriate prescription of effective and safe drugs is the outcome of several variables: adherence to scientifically sound treatment guidelines, availability of quality-assured drugs, accurate assessment of the drug susceptibility testing for the collected Mtb isolates, and adequate follow-up (which depends on the efficiency of the national and regional healthcare infrastructure and of the national TB program).

However, patient's adherence to the prescribed regimens is key, especially for individuals infected by MDR MTB strains, where the duration of the therapy is longer (~24 months) and characterized by a high risk of drug-related adverse events (the currently available therapeutic armamentarium is less effective, less safe, and more expensive if compared with that adopted for drug-susceptible TB) (Nahid et al., 2019; Dheda et al., 2019).

The poor adherence and the risk of adverse events depend on the anti-TB drugs themselves and on their pharmacological interactions with other concomitant therapies.

Then, the clinical success inevitably passes through the prevention of DDIs and drug-adverse events to improve TB treatment adherence and,ultimately, the WHO outcome treatment success. Careful evaluation of possible DDIs before creating an anti-TB regimen is a key moment to ensure efficacy, safety, and ameliorate patients’ quality of life (19,57). Up-to-date knowledge of anti-TB drugs PK/PD parameters coupled with TDM are helpful tools to guide physicians to tailored and effective treatments.

The aim of the present article was to give a concise summary that can aid physicians in their daily clinical practice.

However, several potential interactions are unknown because some of them occurred in countries where an effective pharmaco-vigilance system is not in place. The low TB incidence countries characterized by a lower treatment prescription rate cannot assess some interactions which can occur where more TB patients are treated.

Furthermore, the evolving drug market has a marginal impact in low income countries where TB incidence is high. The combination of those epidemiological conditions does not help identifying the full pharmacological profile of the anti-TB drugs.

More information can be retrieved from the HIV/AIDS-related evidence: major efforts have been performed since the 90s’ to better describe the characteristics of the anti-HIV drugs (Bisson et al., 2020). The high incidence of TB/HIV co-infection has favoured the study of DDIs between the options prescribed for the HIV infection and those for the TB disease.

However, TB patients can have several comorbidities needing complicated drug prescriptions: diabetes mellitus, cancers, COPD, asthma, auto-immune disorders, and other chronic conditions need to be treated with old-fashioned or new drugs with known and unknown DDIs.

The metabolic interactions can increase or decrease drugs’ levels affecting the therapeutic targets and the clinical outcomes. Some adverse events could change the natural history: for instance, the increased hazard of cardiac events related to the new drug bedaquiline, or the old clofazimine, prescribed with potentially cardio-toxic drugs could increase the risk of death.

The relevant costs associated to the poor clinical outcomes and to the occurrence of DDIs-related adverse events should be kept into careful consideration, especially in countries where the financial issues affect the strength and the efficiency of the health-care system. In theory, those events can be prevented.

More research is needed in this delicate field. The process of research and development does not finish after the market approval by the regulatory agencies. Post-marketing surveillance studies, supported by basic and translational research, could change the scenario, adding key insights to the expected improved management of TB patients.

## Funding/support

None.

## CRediT authorship contribution statement

**Niccolò Riccardi:** Conceptualization, Validation, Writing - original draft, Writing - review & editing. **Diana Canetti:** Data curation, Methodology, Validation, Writing - original draft, Writing - review & editing. **Paola Rodari:** Data curation, Methodology, Validation, Writing - original draft, Writing - review & editing. **Giorgio Besozzi:** Validation, Writing - review & editing. **Laura Saderi:** Data curation, Methodology, Validation, Writing - review & editing. **Marco Dettori:** Validation, Writing - original draft, Writing - review & editing. **Luigi R. Codecasa:** Supervision, Validation, Writing - review & editing. **Giovanni Sotgiu:** Conceptualization, Project administration, Supervision, Validation, Writing - review & editing.

## Declaration of competing interest

The authors declare that they have no known competing financial interests or personal relationships that could have appeared to influence the work reported in this paper.
